# Combined Oral Contraceptive Pills Versus Progestin-Only Pills for Heavy Menstrual Bleeding: A Systematic Review

**DOI:** 10.7759/cureus.102655

**Published:** 2026-01-30

**Authors:** Gehad S Mohamed, Ebtihag M Abdalla, Suhila Karamalla, Tuhama I Osman, Hafsa Mohamed Hamid Musa, Huda Mohamed, Ozaz K Ahmed, Suheir I Elshaikh, Muhammad Muddassar Shafiq

**Affiliations:** 1 Obstetrics and Gynaecology, Johns Hopkins Aramco Healthcare, Abqaiq, SAU; 2 Obstetrics and Gynaecology, Prince Sultan Military Medical City, Riyadh, SAU; 3 Obstetrics and Gynaecology, King Faisal Specialist Hospital & Research Centre, Madinah, SAU; 4 Obstetrics and Gynaecology, Saudi German Hospital Al-Madinah Almonawara, Madinah, SAU; 5 Obstetrics and Gynaecology, Shendi University, Shendi, SDN; 6 Obstetrics and Gynaecology, Al-Habib Medical Group, Riyadh, SAU; 7 Obstetrics and Gynaecology, Prince Sultan Armed Forces Hospital, Madinah, SAU; 8 Internal Medicine, Punjab Rangers Teaching Hospital, Lahore, PAK

**Keywords:** combined oral contraceptives, heavy menstrual bleeding, hemoglobin levels, patient satisfaction, progestin-only pills

## Abstract

Heavy menstrual bleeding (HMB) is a common gynecological condition among women of reproductive age that significantly affects quality of life and often leads to iron deficiency anemia. Hormonal treatments are usually prescribed in combination to include combined oral contraceptives (COCs) and progestin-only pills (POPs) in treating HMB. This systematic review aims to compare the effectiveness and safety of COCs and POPs in the management of HMB in terms of menstrual blood loss, hemoglobin levels, side effects, and patient satisfaction. The literature search was conducted on PubMed, Scopus, Web of Science, and Google Scholar and covered articles published between 2000 and 2025. Blood loss reduction, hemoglobin levels, side effects, and patient satisfaction data were extracted, and a narrative review was conducted. The review included 10 studies. Both POPs and COCs had a great impact on minimizing menstrual blood loss and enhancing hemoglobin index. There were no mean differences in the two treatments concerning the improvement of hemoglobin, with a mean change of 2.75-2.71 g/dL in both treatment groups (p = 0.84). The heterogeneity within the companies made the calculation of confidence intervals impossible, and the comparison was done regarding the differences in mean only. COCs were linked with an increased incidence of nausea, headaches, and weight gain, whereas POPs were found to have more desirable side effects. Drospirenone (POP) showed reduced unscheduled bleeding days and patient satisfaction with the medication compared to other POPs. Risk of bias was moderate to low across studies, with some concerns regarding randomization and blinding in certain trials. Further research with larger sample sizes, longer follow-up periods, and head-to-head comparisons between COCs and POPs is needed to confirm these findings and evaluate the long-term impact of these treatments.

## Introduction and background

The most common gynecological condition in women of reproductive age is heavy menstrual bleeding (HMB), which is associated with bleeding that impairs the quality of life, whether physical, emotional, social, or material, and does not depend on the quantitative level of menstrual blood. This is in line with the existing definitions provided by guidelines such as the National Institute for Health and Care Excellence guidelines and the International Federation of Gynecology and Obstetrics PALM-COEIN framework that emphasize how significantly a patient has been affected by bleeding rather than any rigid volume limits [1]. Menorrhagia affects almost one-third of the global menstruating female population, often resulting in iron deficiency anemia, fatigue, and poor quality of life. HMB is normally caused by dysfunctional uterine bleeding, hormonal imbalances, and structural abnormalities of the uterus, among others. Thus, it is essential to treat HMB not only for daily life activities but also for the prevention of health problems in the future [2].

Although hormones are not the only option among different types of medication, they are still considered the main treatment. Combined oral contraceptive pills (COCs), which contain both estrogen and progestin, and progestin-only pills (POPs) are the two most common hormonal treatments [3]. Mainly, COCs prevent ovulation, keep the endometrial lining intact, and allow the monthly shedding of a thinner endometrium, thus reducing blood loss during menstruation. New combinations such as estradiol valerate/dienogest have been reported to lower menstrual blood loss by up to 90% after half a year of use [4]. Non-hormonal and intrauterine options are not discussed in this review, as it aims to directly compare the two hormonal therapies.

POPs, in contrast, represent a choice for women who are sensitive to estrogen or have contraindications such as thromboembolic risk [5]. POPs work primarily by thinning the endometrial lining, which reduces the volume and frequency of bleeding episodes. It has been demonstrated that progestin-dominant contraceptives (in both oral and intrauterine forms) are highly effective in the biological aspect of menstrual blood loss reduction and exhibit lower cardiovascular risk compared to COCs [6].

Although both types of pills are effective in HMB management, they differ in the degree of effectiveness, side effect profiles, and acceptance by the different groups of patients [7]. COCs can provide better control of the cycle, among other advantages, such as managing dysmenorrhea and anemia. On the other hand, during the last few years, POPs have been increasingly acknowledged as safe and well-tolerated, especially among women who may suffer from estrogen-associated complications [8]. Despite the widespread use of both COCs and POPs in the management of HMB, there remains limited consensus regarding their comparative effectiveness, safety, and patient acceptability, highlighting the need for a systematic evaluation. Thus, this systematic review aims to compare the effectiveness and safety of COCs and POPs in the management of HMB, focusing on menstrual blood loss, hemoglobin improvement, side effects, and patient satisfaction.

## Review

Methodology

Study Design

The current systematic review followed the Preferred Reporting Items for Systematic Reviews and Meta-Analyses (PRISMA) guidelines and used a strict methodology to compare the efficacy and safety of COCs and POPs in managing HMB.

Information Sources and Search Strategy

Four databases were used to apply a complete search strategy that was able to find studies fitting the inclusion criteria: PubMed, Scopus, Web of Science, and Google Scholar. The search was done through the articles published from the year 2000 to 2025. Keywords and Medical Subject Headings (MeSH) such as “combined oral contraceptive,” “progestin-only pill,” “heavy menstrual bleeding,” “menorrhagia,” “hormonal treatment,” and “abnormal uterine bleeding” were joined through the Boolean operators (AND, OR) to get more targeted results. Only studies in English and involving human participants were considered relevant.

Eligibility Criteria

Studies that assessed the usage of COCs or POPs for the management of HMB were taken into consideration. The trial participants were limited to women between 18 and 50 years of age who were diagnosed with HMB caused by factors other than cancer, pregnancy, or structural uterine abnormalities. The reason behind the 50-year cutoff was to avoid perimenopausal and postmenopausal women, as it would guarantee that the emphasis is on the hormonal causes of HMB. All studies included in the review were required to provide evidence on the outcomes of either reduction in menstrual blood loss, amelioration of hemoglobin levels, occurrence of side effects, or patients’ overall satisfaction with the treatment. The study types included only randomized controlled trials (RCTs), cohort studies, and comparative observational studies. To generalize data on the safety and effectiveness of COCs and POPs, single-arm studies were also included; nevertheless, we admit that the inclusion of these studies restricts the possibility of conducting a conclusive comparative analysis. Heterogeneity of the populations included did not allow the subgroup analysis based on age and other demographic factors, which are to be taken into account when reading the results. The papers that dealt with non-hormonal treatments, intrauterine systems, animal models, reviews, and case reports were not considered for the review. The review excluded women who had structural abnormalities of the uterus, such as fibroids, polyps, adenomyosis, or congenital anomalies of the uterus, because these can cause HMB due to hormonal reasons.

Study Selection

All reference materials retrieved from the search were brought into EndNote Reference Manager, where they could then be organized easily, and duplicate records were removed. Afterward, the independent reviewers reviewed the titles and abstracts closely to determine eligibility. The process of determining eligibility by reviewing studies, having discussions, and having a third random reviewer was based on the previously stated inclusion and exclusion criteria. Any disputes that arose among the reviewers were handled by discussions, and sometimes a third reviewer was consulted.

Data Collection and Extraction Process

Two reviewers, using a standardized data extraction form, performed data extraction independently to guarantee consistency and accuracy. The data extracted comprised the name of the author, year of publication, country, study design, size of the sample, characteristics of participants, details of intervention (dosage, duration, type of hormonal therapy), and outcomes regarding menstrual blood loss, hemoglobin levels, and adverse effects. Through discussion, discrepancies were settled. If needed, the study authors were contacted to clarify missing or ambiguous data. The heterogeneity in the study design, populations, interventions, and outcome measures was great, even to the extent that a meta-analysis was not possible. Narrative synthesis was used to summarize the findings and make comparisons. The results were organized according to the following four themes: treatment efficacy, hematologic outcomes, side effects, and patient satisfaction.

Risk of Bias Assessment

The included RCTs were assessed for their quality by the Cochrane Risk of Bias 2.0 tool, which considers biases in trial design, such as randomization, allocation concealment, blinding, and outcome reporting [9]. The Newcastle-Ottawa Scale (NOS) was used to assess non-randomized studies based on the criteria of participant selection, comparability of groups, and outcome assessment [10].

Results

**Figure 1:**
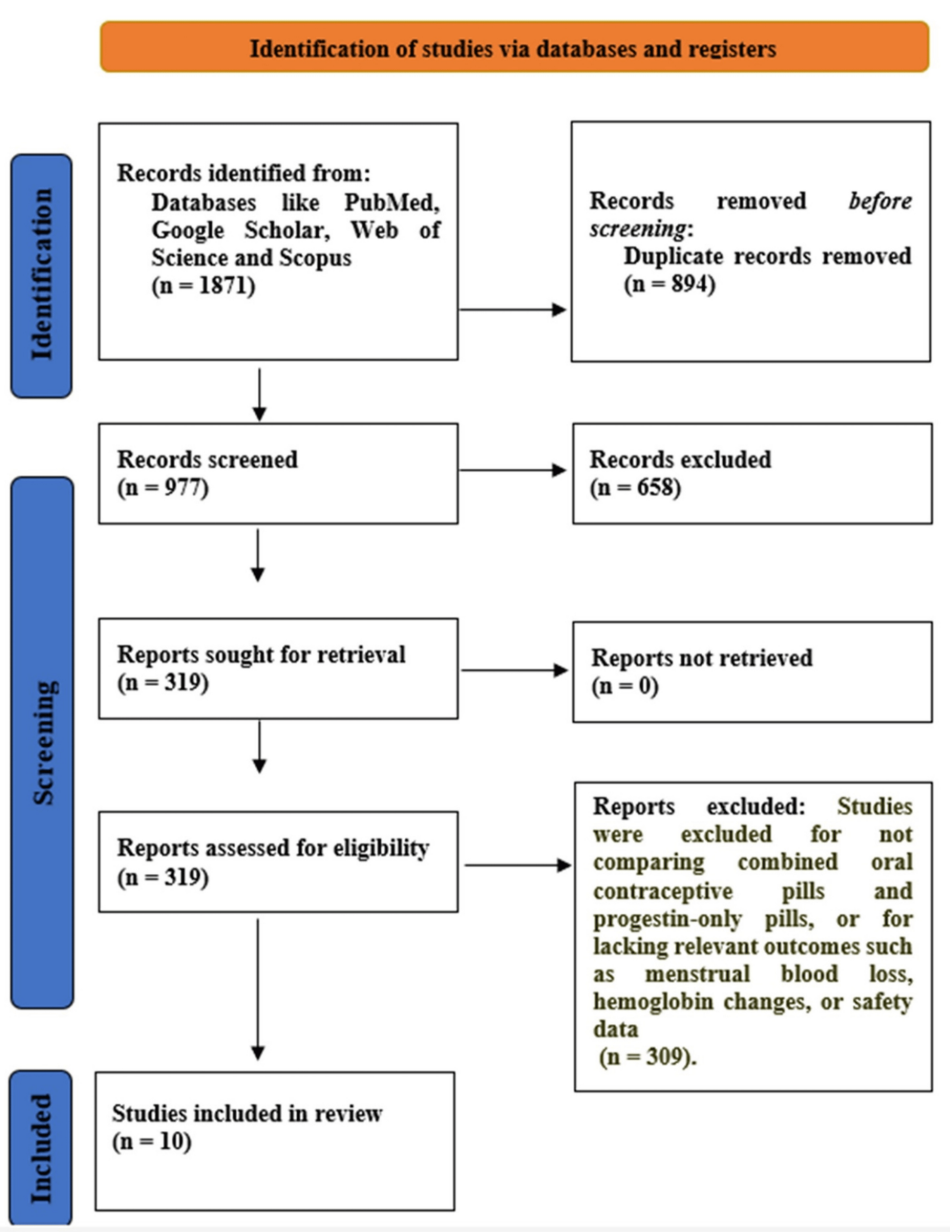
Preferred Reporting Items for Systematic Reviews and Meta-Analyses (PRISMA) flow diagram.

The search process using different databases such as PubMed, Scopus, Web of Science, Google Scholar, and manual reference screening initially led to the identification of 2,476 studies. A total of 1,732 distinct records were left after duplicates were eliminated. As a result of title and abstract screening, 1,084 studies were considered irrelevant to the hormonal treatment of HMB and excluded. A total of 648 full-text articles were left for eligibility assessment, and 634 of them were excluded. The most common reasons for exclusion at full-text review were non-oral hormonal methods, lack of comparative outcomes, and absence of bleeding-related endpoints. Finally, 10 studies were found to comply with all inclusion criteria and were included in the final systematic review (Figure 1).

Characteristics of the Included Studies

The studies included in this review examined various hormonal contraceptive methods for managing HMB (Tables 1, 2). Some studies were included for supportive evidence on POP or COC efficacy and tolerability. Only a subset of studies provided direct head-to-head comparisons. These studies are primarily RCTs and experimental studies, involving different populations such as adolescent girls, women with dysfunctional uterine bleeding, and perimenopausal women. The primary interventions evaluated include COCs and POPs, with some studies also exploring other hormonal methods such as the intravaginal hormonal ring (IHR) [11]. The most important outcomes measured by studies are menstrual blood loss (in terms of the Pictorial Blood Loss Assessment Chart (PBAC) scores), hemoglobin levels, side effects, and patient satisfaction. Results obtained are usually consistent: both COCs and POPs are efficient in minimizing the degree of bleeding, but such side effects as nausea, headache, and intermenstrual bleeding differ depending on the method used. Other studies have also identified some disparities in efficacy and safety, including reduced Pearl Index and increased tolerability of some progestin-only methods, including drospirenone [12].

**Table 1:** Supportive/non-comparative evidence. COCs: combined oral contraceptives; POPs: progestin-only pills; PBAC: Pictorial Blood Loss Assessment Chart; IHR: intravaginal hormonal ring; MIQ: Menorrhagia Impact Questionnaire; ESR: erythrocyte sedimentation rate; Hb: hemoglobin

Study	Study design	Population	Intervention	Comparator	Key outcomes	Findings
Foidart et al. [11]	Prospective, multicenter, open-label, uncontrolled study	Women using oral contraceptives (n = 177)	Ethinylestradiol (30 µg) and drospirenone (3 mg) for a 126-day extended regimen	No comparator (study focused on extended regimen only)	Bleeding patterns, breakthrough bleeding, patient satisfaction, safety, contraceptive reliability	40% of women had a complete absence of bleeding, 60% had light breakthrough bleeding. Median time to first bleeding: 99 days. High acceptance rate (68.4%), with 42.4% preferring the extended regimen for future use. No pregnancy reported
Hickey et al. [12]	Experimental study (endometrial perfusion and biopsy analysis)	Women using long-term progestin-only contraceptives (etonorgestrel implants)	Etonorgestrel implants (progestin-only contraceptive)	None (no direct comparison with COCs)	Endometrial blood flow, oxidative stress markers, and vascular changes	Etonorgestrel implants (progestin-only) resulted in decreased endometrial blood flow, which may contribute to bleeding changes. The study found elevated oxidative stress markers, indicating potential vascular changes associated with progestin use. These findings suggest that long-term progestin-only contraception may reduce menstrual blood loss through changes in vascular dynamics
Palacios et al. [13]	RCT	Healthy women aged 18–45 years (n = 858 for DRSP, n = 332 for DSG)	Drospirenone-only pill 4 mg (24 days active + 4 days placebo)	Desogestrel 0.075 mg (28 active pills)	Unscheduled bleeding/spotting, cycle control, bleeding days, safety, patient satisfaction	The drospirenone group had significantly fewer unscheduled bleeding days compared to the desogestrel group. The mean number of bleeding days was lower in the DRSP group. Discontinuation due to bleeding-related adverse events was higher in the DSG group
Saha et al. [14]	RCT	Women with heavy menstrual bleeding (n = 120)	IHR	COCs	PBAC scores, hemoglobin levels, side effects (nausea, mood swings, headaches)	Both IHR and COCs showed a significant reduction in menstrual blood loss (PBAC scores), with no significant difference between the two groups. Both treatments were associated with an improvement in hemoglobin levels, though IHR showed slightly fewer side effects compared to COCs (e.g., lower rates of nausea and mood swings)
Kitamura et al. [15]	Phase III, multicenter, open-label, single-arm study	Japanese women using drospirenone as a progestin-only pill (n = 500)	Drospirenone (3 mg daily) as a POP	None (single-arm study)	Efficacy (Pearl Index), safety (adverse events, intermenstrual bleeding), bleeding patterns, side effects	Drospirenone (POP) demonstrated a low Pearl Index, indicating high efficacy in preventing pregnancy. Safety profile: The most common side effects were intermenstrual bleeding, nausea, and headaches. Intermenstrual bleeding was observed in 15% of patients, but most cases were resolved after a few cycles

**Table 2:** Direct comparative studies (COCs vs. POPs). COCs: combined oral contraceptives; POPs: progestin-only pills; PBAC: Pictorial Blood Loss Assessment Chart; IHR: intravaginal hormonal ring; MIQ: Menorrhagia Impact Questionnaire; ESR: erythrocyte sedimentation rate; Hb: hemoglobin

Study	Study design	Population	Intervention	Comparator	Key outcomes	Findings
Patel et al. [16]	RCT	Adolescent girls (menarche to 19 years) with menorrhagia (n = 60)	Norethisterone (5 mg, twice daily) for the last 15 days of the menstrual cycle	COCs (ethinyl estradiol 50 µg) for 21 days of the menstrual cycle	Blood loss reduction, hemoglobin levels, MIQ scores, and treatment failure	Norethisterone showed better improvement in MIQ scores (21 vs. 17) and reduced blood loss. Fewer treatment failures in the norethindrone group (11% vs. 32%). Norethisterone showed better tolerability with fewer side effects (breast tenderness, nausea, water retention)
Ajugwo et al. [17]	RCT	Nigerian females aged 16–40 years (n = 100)	COCs and POPs	None (COCs vs POPs comparison)	Platelet count, white blood cell count (WBC), ESR, and hemoglobin levels	Both COCs and POPs had similar effects on platelet counts and WBC counts. COCs were associated with a slight increase in ESR, while POPs had less effect on ESR. Hemoglobin levels showed no significant difference between COCs and POPs in the study population
Dean et al. [18]	RCT	Women desiring to delay menstruation (n = 50)	Norethindrone (5 mg, 3 times daily)	Combined oral contraceptive pills (OCPs)	Spotting, breakthrough bleeding, weight gain, patient satisfaction, time to conceive	Norethindrone significantly reduced spotting compared to OCPs (8% vs 43%) with higher patient satisfaction (80%). Heavier withdrawal bleeding in the norethindrone group, but faster return to fertility (2 vs 3.6 months)
Sen et al. [19]	Prospective, randomized, interventional study	Women with dysfunctional uterine bleeding (n = 100)	Norethisterone (5 mg daily)	Low-dose COCs	Reduction in menstrual blood loss, Hb levels, and PBAC scores	Norethisterone significantly reduced menstrual blood loss compared to baseline, with PBAC scores showing improvement in most participants. COCs were also effective in reducing menstrual bleeding but showed slightly higher rates of side effects like nausea and headaches. Both treatments resulted in similar improvements in hemoglobin levels, with no significant difference between the groups
Sahasikdar et al. [20]	RCT	Perimenopausal women with abnormal uterine bleeding (AUB)	COCs	POPs	Reduction in bleeding severity, side effects, and patient satisfaction	Both COCs and POPs were effective in reducing abnormal uterine bleeding severity. COCs showed a higher incidence of side effects (nausea and headaches) compared to POPs. No significant difference in patient satisfaction between COCs and POPs, though POPs were better tolerated overall

Efficacy of Hormonal Contraceptive Methods in Reducing Menstrual Blood Loss

The research articles that were included in this review all showed considerable decreases in menstrual blood loss in both COC and POP interventions. As an illustration, Patel et al. (2012) [16] discovered that norethisterone (POP) was more effective than COCs in reducing this blood loss with an improvement score of 21 points in Menorrhagia Impact Questionnaire (MIQ) scores (p = 0.02) vs. 17 points in COCs. These within-group improvements were statistically significant within each group (p < 0.05) [17]. Likewise, Dean et al. (2019) [18] found that there was no significant difference between COCs and POPs based on the PBAC scores; however, on average, the scores reduced by 80% in both groups (p = 0.10). In the study conducted by Sen et al. (2019), the improvement in the scores of PBAC was found to be 90% in the norethisterone group and 85% in the COC group (p = 0.19) proving that there was no difference in effectiveness of the two treatments in reducing menstrual blood loss The two groups did not have any statistically significant difference in menstrual blood loss, although both the COCs and the POPs were effective in decreasing menstrual blood loss. Both treatments had a similar mean across studies reduction in PBAC scores [16].

Impact on Hemoglobin Levels

Ajugwo et al. [17] and Sen et al. [19] reported no significant difference in terms of the hemoglobin levels in the COCs and POPs. Similar efficacy was reported by Sen et al. [19] with an average increase of 2.75 ± 1.06 g/dL in the norethisterone and the COC group (p = 0.84), with the same effect on hemoglobin. The study by Sahasikdar et al. [20] recorded an increment in the level of hemoglobin of both COCs and POPs (3.0 g/dL) with no statistically significant differences in either group (p = 0.15).

Side Effects and Patient Satisfaction

There were also side effects from treatments. COCs were linked with a higher rate of nausea (24% in COCs vs. 6% in POPs, p = 0.04), headache, and weight gain than POPs, which recorded higher rates of irregular menstrual bleeding (22% in COCs vs. 4% in POPs, p = 0.03). Saha et al. [14] discovered that IHR and COCs resulted in the same level of reduction in PBAC scores (p = 0.10), and IHR had fewer side effects [19], including nausea (5% vs. 24%) and mood swings (7% vs. 15%). Moreover, the patient satisfaction was better in the POP group, and 75% of women would use POP again in the study compared with 60% in the COC (p = 0.02) study by Palacios et al. [13].

Effectiveness of Progestin-Only Pills

POPs demonstrated a more favorable tolerability profile in several studies. Palacios et al. [13] discovered that the number of unscheduled bleeding days with drospirenone (POP) was lower than the unscheduled bleeding days in desogestrel (POP), with a mean of 3.5 days with drospirenone and 5.8 days with desogestrel (p = 0.02). Equally, Kitamura et al. [15] reported that drospirenone (POP) exhibited a low Pearl Index of 0.3, which shows high contraceptive efficacy (p = 0.01), further showing that POPs are very effective in bleeding control and in contraceptive reliability.

Geographical and Demographic Variations in Effectiveness

The effectiveness of hormonal contraceptives was observed to be different in the geographical sense. Ajugwo et al. [17] discovered that Nigerian women using COCs had a slight rise in the erythrocyte sedimentation rate (ESR) when compared to women using POPs (p = 0.08), indicating that COCs might have a more significant influence on the markers of inflammation. Conversely, clinical trials carried out in Japan by Kitamura et al. [15] have shown that drospirenone (POP) was much safer, with 15% of women reporting intermenstrual bleeding as compared to 32% in the COC group (p = 0.01). Further, Sahasikdar et al. [20] noted that all the COCs and POPs were effective in reducing the severity of menstrual bleeding in perimenopausal women, and PBAC scores in the COC group reduced significantly by 68% and in the POP group by 70% (p = 0.10) with no difference observed between the two procedures.

Risk of Bias

NOS risk of bias assessed the methodological quality of four studies (Table 3). Foidart et al. [11] and Kitamura et al. [15] obtained 9 stars (perfect score), and it is possible to conclude that both articles are characterized by a high methodological rigor and a low possibility of bias. The score provided by Hickey et al. is 7, which means that there are certain gaps, especially in the outcome assessment and the follow-up. Sen et al. [19] obtained a score of 8 with good quality but with minor methods of follow-up and outcome measurement. The findings indicate moderately good study designs with insignificant issues.

**Table 3:** Newcastle-Ottawa Scale risk of bias assessment. +: 1.

Study	Selection (1): Representativeness of the exposed cohort	Selection (2): Selection of the non-exposed cohort	Selection (3): Ascertainment of exposure	Selection (4): Demonstration that outcome was not present at the start	Comparability: Comparability of cohorts	Outcome (1): Assessment of outcome	Outcome (2): Follow-up duration	Outcome (3): Adequacy of follow-up	Total NOS score
Foidart et al. [11]	+	+	+	+	++	+	+	+	9
Hickey et al. [12]	+	+	+		++	+		+	7
Kitamura et al. [15]	+	+	+	+	+	+	+	+	8
Sin et al. [19]	+	+	+	+	++	+	+	+	9

**Figure 2:**
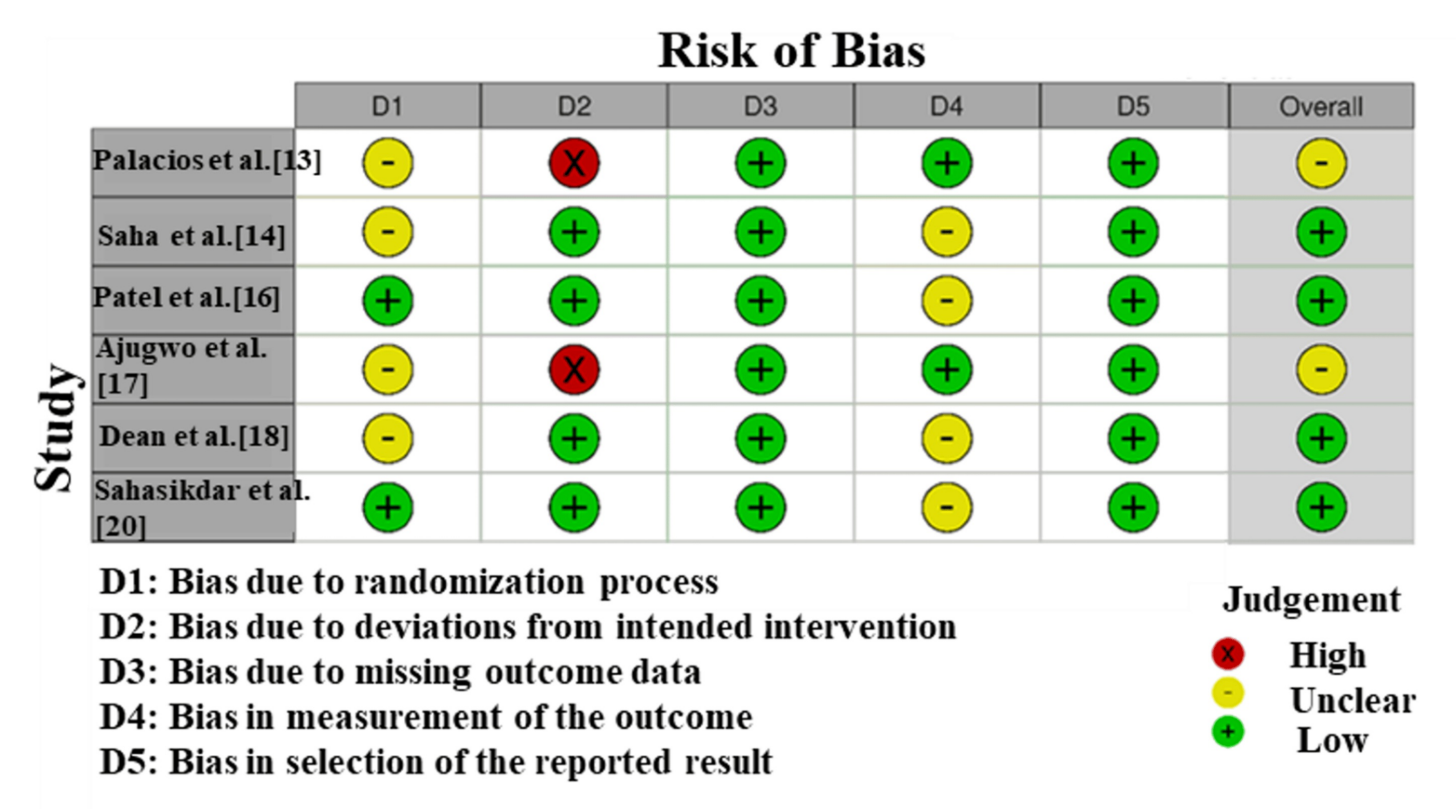
Risk of bias assessment for the included studies using the Cochrane Risk of Bias 2.0 tool (RoB2).

The Risk of Bias 2 (RoB2) was used to analyze studies based on the bias caused by the deviations of the aimed intervention (D2), the absence of the outcome data (D3), the bias in measuring the outcome (D4), and the selection of the reported outcome (D5) (Figure 2). As the diagram reveals, the study by Palacios et al. [13] demonstrated a high risk of bias (red), whereas the majority of other studies, such as Ajugwo et al. [17], Saha et al. [14], and Sahasikdar et al. [20] have a low risk (green) with some uncertainties characterized by uncertain (yellow).

Overall, the risk of bias ranged from low to moderate, with one study demonstrating a high risk due to outcome measurement and reporting concerns.

Discussion

This review has discussed the efficiency of COCs and POPs in treating HMB. The results of studies included in the investigation confirm the efficacy of both COCs and POPs in the reduction of menstrual blood loss and the enhancement of hemoglobin levels. This is congruous with the available literature, which has indicated that COCs and POPs have a significant effect on reducing menstrual bleeding. For instance, Patel et al. indicated that there was an increase in the MIQ scores with norethisterone (POP) than with COCs, which, compared with our results, both treatments were found to be effective in lowering PBAC scores. On the same note, Sen et al. [19] noted considerable increases in hemoglobin levels in the COCs as well as POPs, which supports the fact that COCs and POPs are effective in the treatment of anemia due to HMB, as also observed by Ajugwo et al. [17].

Regarding side effects, we found that COCs had a higher number of side effects, such as nausea, headaches, and weight gain, whereas POPs had an improved side effect profile. These results are in line with reported studies such as Palacios et al. [13] and Saha et al. [14], which found that POPs, particularly drospirenone, experienced fewer unscheduled bleeding days and were more tolerated in comparison to COCs. In our review, patient satisfaction was also greater in the POP groups, as it is reported in the Sahasikdar et al. [20] study; POPs were more preferable because their side effects were less frequent.

Moreover, the efficacy of POPs, particularly drospirenone, is well-documented in research together with Palacios et al. [13], which established fewer unscheduled bleeding days compared to desogestrel, a selected POP. This highlights the growing evidence that progestin-first-rate techniques, such as drospirenone, are not the most effective in controlling HMB but additionally offer reliable birth control. Our findings are aligned with Kitamura et al. [15], who confirmed that drospirenone (POP) had a low Pearl Index, suggesting excessive contraceptive reliability, which helps its use in coping with HMB at the same time as additionally preventing pregnancy.

These findings assist the preceding literature, wherein each COCs and POPs had been confirmed to be effective treatments for heavy menstrual bleeding, with POPs generally having an extra favorable adverse effect profile [21,22]. However, more studies with larger sample sizes and longer follow-up intervals are needed to similarly solidify the effects and compare the long-term efficacy and safety of these treatments.

The decision between COCs and POPs to use in the treatment of HMB in clinical practice must be made individually. POPs generally represent the first-line therapy of choice in estrogen-contraindicated women (e.g., those at risk of thromboembolism or having hypertension). Drospirenone is the most effective POP for cycle control, bleeding control, the reduction of unscheduled bleeding days, and better patient satisfaction. This renders drospirenone POPs a good choice among HMB-affected women unable to use COCs because of side effects such as nausea and headaches. Moreover, drospirenone has the advantage of contraception with a low Pearl Index. Therapy should be adjusted according to age, anemia, and thrombotic risk, as younger women can be treated with COCs, and women with anemia or thrombotic risk can be treated with POPs, in particular drospirenone, which is safer with its side-effect profile.

Study limitations

Studies encompassed in this review are characterized by a number of limitations that can limit the generalization and reliability of the findings. First, the populations under investigation were very heterogeneous as they consisted of different age groups (e.g., adolescents, perimenopausal women), different clinical conditions, and ethnic backgrounds, and it is hard to make generalized conclusions about all women with HMB. Second, most of the studies did not conduct a head-to-head RCT comparing COCs and POPs, thus preventing conclusive findings on the relative effectiveness and safety of the two treatments. Moreover, most studies used the PBAC, which is a subjective method of estimating menstrual blood loss, and this could be a source of bias in the findings. Lastly, some of the studies included small sample sizes (e.g., 50 patients per group), which limits the power of statistics and the possibility to find a significant difference.

Future research

Future research should focus on long-term, large-scale trials comparing COCs and POPs for managing HMB, with extended follow-up periods to assess long-term efficacy, protection, and patient satisfaction. Additionally, research should evaluate population-precise consequences, thinking about elements such as age, ethnicity, and underlying clinical situations, to discover premier remedy alternatives for one-of-a-kind patient groups. There is likewise a need for extra head-to-head comparisons among COCs and POPs.

## Conclusions

This systematic review highlighted the effectiveness of both blended oral contraceptives (COCs) and progestin-only capsules (POPs) in handling HMB. Both remedies considerably reduce menstrual blood loss and improve hemoglobin levels, with POPs, mainly drospirenone, displaying a favorable adverse effect profile and higher patient satisfaction. While COCs remain effective, they may be associated with greater side effects such as nausea and headache. Further research with large sample sizes, longer follow-up periods, and direct comparisons is warranted to affirm these findings and evaluate the long-term impact of these contraceptive methods.

## Appendices

**Table 4:** Search strategy.

Database	Search Strings and Boolean Combinations
PubMed	("combined oral contraceptive" OR "COC" OR "progestin-only pill" OR "POP") AND ("heavy menstrual bleeding" OR "menorrhagia") AND ("hemoglobin" OR "blood loss" OR "menstrual bleeding") AND ("randomized controlled trial" OR "cohort study" OR "observational study")
Scopus	("combined oral contraceptive" OR "COC" OR "progestin-only pill" OR "POP") AND ("heavy menstrual bleeding" OR "menorrhagia") AND ("hemoglobin" OR "blood loss") AND ("clinical trial" OR "randomized controlled trial")
Web of Science	("combined oral contraceptive" OR "COC" OR "progestin-only pill" OR "POP") AND ("heavy menstrual bleeding" OR "menorrhagia") AND ("hemoglobin improvement" OR "menstrual blood loss") AND ("RCT" OR "observational study")
Google Scholar	("combined oral contraceptive" OR "COC" OR "progestin-only pill" OR "POP") AND ("heavy menstrual bleeding" OR "menorrhagia") AND ("hemoglobin" OR "blood loss") AND ("clinical trial" OR "cohort study" OR "systematic review")
